# Comprehensive Characterization and Prognostic Modeling of Efferocytosis-Related Genes in Cutaneous Melanoma

**DOI:** 10.7150/jca.130446

**Published:** 2026-07-29

**Authors:** Yuliang Sun, Zhihu Ma, Yanlin Li, Anhao Shi, Gang Wang

**Affiliations:** Department of Orthopedics, Qilu Hospital of Shandong University, Jinan, China, 250000.

**Keywords:** cutaneous melanoma, efferocytosis, immune infiltration, machine learning, TTYH3

## Abstract

**Background:**

Cutaneous melanoma (CM) is a highly aggressive skin cancer with poor prognosis in advanced stages. Efferocytosis, the process by which apoptotic cells are cleared by phagocytes, plays a dual role in tumor immunity and progression. However, the comprehensive role of efferocytosis-related genes (EFRGs) in CM remains unclear.

**Methods:**

We conducted a multi-omics analysis by integrating transcriptomic data from TCGA, GEO and GTEx databases, identifying differentially expressed genes and performing WGCNA to define EFRG signatures. Based on the expression profiles of differentially expressed EFRGs, we identified molecular subtypes, evaluated immune infiltration using ESTIMATE and ssGSEA. A prognostic EFRG risk scoring model was further constructed and validated using multiple machine learning algorithms. Western blot, CCK8 assay, colony formation assay and Transwell assays were performed to validate the functional role of the screened EFRG.

**Results:**

21 DE-EFRGs with significant prognostic value were identified and three distinct molecular subtypes were subsequently defined. The EFRG-based scoring model effectively stratified patients into high- and low-risk groups with distinct survival outcomes and immune microenvironment characteristics. The low-risk group exhibited a more immune-activated phenotype and greater sensitivity to immunotherapy and chemotherapeutic agents.* In vitro* functional validation revealed that knockdown of one screened EFRG, TTYH3, significantly inhibited melanoma cell proliferation and migration.

**Conclusions:**

Our study comprehensively characterizes EFRG patterns in CM and proposes a robust EFRG-based prognostic model. *TTYH3* is identified as a potential oncogenic effector and therapeutic target. These findings may guide personalized immunotherapy and improve prognostic assessment in melanoma.

## Introduction

Cutaneous Melanoma (CM) is a highly aggressive form of skin cancer originating from melanocytes and is associated with a high risk of metastasis and poor prognosis in advanced stages 1. Despite recent advances in targeted therapies and immunotherapies, such as BRAF/MEK inhibitors and immune checkpoint inhibitors (ICIs), clinical outcomes remain heterogeneous among patients, with varying responses and survival rates 2-4. Therefore, further risk stratification is essential to identify patients at higher risk of disease progression or treatment resistance, enabling more personalized therapeutic strategies and improved prognostic assessment 5. Notably, CM is characterized by a high cellular turnover rate and frequent apoptosis induced by both intrinsic tumor stress and therapeutic interventions, particularly immunotherapy and targeted therapy 6. Dysregulation of the efficient clearance of tumor cells may profoundly influence immune activation, antigen presentation, and tumor progression.

Efferocytosis is a tightly regulated and highly efficient process by which apoptotic cells are recognized and cleared by professional phagocytes, such as macrophages and dendritic cells 7. Several key steps are involved in this process: the release of "find-me" signals by apoptotic cells to recruit phagocytes, recognition of "eat-me" signals and engagement of specific receptors on phagocytes that mediate engulfment 8. Efferocytosis not only ensures the removal of dying cells without triggering inflammation but also promotes an anti-inflammatory and pro-resolving microenvironment through the secretion of cytokines such as IL-10 and TGF-β 9, 10. Impaired or inefficient efferocytosis can lead to secondary necrosis of apoptotic cells, contributing to chronic inflammation, autoimmune responses, and tumor progression 11, 12. Since immune surveillance plays a central role in CM carcinogenesis, understanding the molecular mechanisms and regulatory pathways of efferocytosis is critical for developing therapeutic strategies targeting diseases associated with defective cell clearance.

Chronic, low-grade inflammation is a contributing factor in the tumorigenesis process 13, 14. Evidence from both murine models and patient samples with autoimmune diseases has demonstrated that efferocytosis plays a role in the formation of chronic inflammatory pathological states 15-17. In the tumor microenvironment (TME), efferocytosis is often aberrantly activated. As apoptotic tumor cells accumulate, this process accelerates the clearance of apoptotic debris, thereby preventing its conversion into pro-inflammatory necrosis and reducing the immune system's ability to recognize and eliminate tumor cells 18. Moreover, efferocytosis-associated signaling pathways, such as those involving TAM receptors, MerTK and Gas6, can promote the polarization of macrophages toward the M2 phenotype, leading to the secretion of anti-inflammatory cytokines. This fosters an immunosuppressive "cold tumor" environment, facilitating tumor immune evasion, sustained growth, and metastatic dissemination 19, 20. Tumor cells may also manipulate the function of immune cells by regulating efferocytosis-related receptors 21, 22. Therefore, targeting key regulators of efferocytosis may represent a potential strategy to improve the tumor immune microenvironment and enhance the efficacy of immunotherapy, warranting further investigation 23.

In the TME, efferocytosis is often aberrantly activated due to the accumulation of apoptotic tumor cells, resulting from rapid proliferation and therapeutic stress 12. In melanoma, which is known for its high mutational burden and strong immunogenicity, efficient immune activation is critical for tumor control and response to immunotherapy 1. However, excessive or dysregulated efferocytosis may dampen anti-tumor immunity by promoting macrophage polarization toward an immunosuppressive M2 phenotype and reducing the release of danger-associated molecular patterns (DAMPs), thereby impairing immune recognition 19. Moreover, key efferocytosis-related signaling pathways, including TAM receptors such as MerTK and their ligand Gas6, have been implicated in melanoma progression and immune evasion 24. These findings suggest that efferocytosis is not merely a bystander process but may actively shape the immunological landscape of CM. Given the central role of immune regulation in CM progression and therapy response, and the critical function of efferocytosis in modulating immune homeostasis and inflammation 25, we hypothesized that efferocytosis-related genes (EFRGs) may represent key determinants of tumor behavior and patient prognosis in CM. However, its global molecular characteristics, prognostic value, and immunological implications in CM remain poorly defined.

In this study, we performed a comprehensive multi-omics analysis, incorporating differential expression analysis, weighted gene co-expression network analysis (WGCNA), molecular subtyping, immune infiltration assessment, and machine learning modeling, to explore the expression patterns, prognostic significance, and immune microenvironment associations of EFRGs in CM. Furthermore, *TTYH3*, identified as a key EFRG, was found to be aberrantly expressed in melanoma cells and to promote melanoma cell proliferation and invasion *in vitro*, partially validating the reliability of the bioinformatics findings. This study aims to provide novel biomarkers and theoretical support for precision immunotherapy and risk stratification in melanoma patients.

## Materials and Methods

### Data Collection

In this study, transcriptomic data and baseline clinical information for normal control (NC) samples and CM samples were obtained from publicly available databases. Specifically, transcriptomic matrix data of normal skin tissues were retrieved from the UCSC Xena database and used as controls. Corresponding transcriptomic profiles and clinical data for CM samples were acquired from The Cancer Genome Atlas (TCGA) database. Transcriptomic matrices were preprocessed, and gene identifiers were annotated using Perl scripts. After excluding samples lacking complete clinical prognosis data, a total of 813 NC samples and 447 CM samples were included for further analysis. Batch effect correction and normalization between datasets were performed using the "sva" package in the R environment. In addition, an independent external validation cohort consisting of 207 CM samples with complete survival data was obtained from the Gene Expression Omnibus (GEO) under the accession number GSE65904. Gene annotation was conducted using Perl scripts according to the platform annotation file (GPL10558: Illumina HumanHT-12 V4.0 expression beadchip). Furthermore, somatic mutation data (TMB) and copy number variation (CNV) data for CM samples were also retrieved from the TCGA database.

### Differential Expression Analysis and WGCNA Model Construction

Differentially expressed genes (DEGs) between NC and CM samples were identified using the thresholds of |fold change| ≥ 1.5 and adjusted *p*-value < 0.05. The “ggplot2” package in R was used for visualization. GSEA was performed using the corresponding R package to assess pathway-level alterations based on gene expression fold changes. WGCNA was then conducted using the “WGCNA” R package to identify gene modules associated with clinical traits. Initially, hierarchical clustering was performed to detect and remove outlier samples. Following sample quality control, a soft threshold power (β = 8) was selected to construct a scale-free topological network (R² = 0.85). Gene modules were identified through dynamic tree cutting and subsequently merged based on module similarity. Pearson correlation analysis was used to evaluate the associations between gene modules and clinical traits, and key modules were selected based on the strength of correlation coefficients. The relationship between module membership (MM) and gene significance (GS) was visualized using scatter plots.

### Identification of Differentially Expressed Efferocytosis-Related Genes and Molecular Subtype Characterization

Based on previously published literature, a total of 157 EFRGs were compiled for subsequent analysis (Supplementary Table 1). Differentially expressed efferocytosis-related genes (DE-EFRGs) were identified by intersecting the list of 157 predefined EFRGs with DEGs using the thresholds of |log2 fold change| ≥ 0.585 and adjusted *p* < 0.05. Subsequently, genes within clinically relevant WGCNA modules (|correlation coefficient| > 0.5 and *p* < 0.01) were further selected to ensure biological relevance. Finally, univariate Cox regression analysis (*p* < 0.05) was performed to identify prognostically significant DE-EFRGs for downstream analysis. The “Venn” R package was used to identify differentially expressed EFRGs (DE-EFRGs) by intersecting EFRGs with differentially expressed genes between groups. The frequency of CNV in DE-EFRGs was visualized using the “ggplot2” R package. Mutation profiles of DE-EFRGs were displayed using waterfall plots generated with the “maftools” R package. To assess the chromosomal distribution of DE-EFRGs, the “RCircos” R package was employed to construct a genome-wide co-localization plot based on chromosomal locations. To identify molecular subtypes, unsupervised consensus clustering was performed using the “ConsensusClusterPlus” R package with the k-means algorithm, where the number of clusters (k) ranged from 2 to 9. Consensus matrices, cumulative distribution function (CDF) curves, and delta area plots were generated to determine the optimal number of clusters. Principal component analysis (PCA) was conducted using “ggplot2” to visualize the distribution patterns of different subtypes. Kaplan-Meier (KM) survival curves were generated using the “survival” R package and log-rank test to evaluate prognostic differences across molecular subtypes.

### Immune Microenvironment Landscape and Prediction of Immunotherapeutic Response

The ESTIMATE algorithm (Estimation of STromal and Immune cells in MAlignant Tumor tissues using Expression data) was applied using the “estimate” R package to assess the immune microenvironment of each sample. Immune score, stromal score, ESTIMATE score, and tumor purity were calculated accordingly. Based on predefined immune cell marker gene sets, single-sample gene set enrichment analysis (ssGSEA) was performed using the “GSVA” R package to quantify the relative abundance of 23 immune cell types in each sample. Immunophenoscore (IPS) data for CM samples were obtained from The Cancer Immunome Atlas (TCIA), and differences in IPS among molecular subtypes were analyzed using “ggplot2,” to infer potential response to CTLA-4 and PD-1 blockade therapies.

### Construction of the EFRG-Based Prognostic Scoring System Using Integrated Machine Learning Algorithms

To evaluate the prognostic value of DE-EFRGs in CM, univariate Cox regression analysis was conducted using the “survival” R package. An integrated machine learning framework was applied under a leave-one-out cross-validation (LOOCV) strategy, using GSE65904 as an independent external validation cohort. A total of 101 algorithmic combinations derived from 10 machine learning approaches—namely “gbm,” “plsRcox,” “randomForestSRC,” “glmnet,” “superpc,” “CoxBoost,” “survivalsvm,” “mixOmics,” “survcomp,” and “BART”—were constructed. For each model, the concordance index (C-index) was calculated in both the TCGA and GSE65904 cohorts to assess performance. The optimal model combination was used to identify key prognostic EFRGs, which were then incorporated into a multivariate Cox regression model to establish the EFRG-based prognostic scoring index. The optimal model combination was selected based on the highest average C-index across the TCGA and GSE65904 cohorts. Candidate genes were further reduced using algorithm-specific feature selection procedures, and those consistently retained across models were included in a multivariate Cox regression model to construct the final EFRG score. CM patients in both cohorts were stratified into high- and low-score groups based on the median EFRG score. KM survival curves were generated using the “survival” R package, and the log-rank test was applied to compare overall survival between the two EFRG score subgroups.

### Independent Prognostic Analysis and Nomogram Construction

Univariate and multivariate Cox regression analyses were performed using the “survival” R package to calculate the hazard ratios (HR) and corresponding p-values for clinical prognostic variables and EFRG score, thereby assessing their independent prognostic value. Based on the “rms” R package, a nomogram was constructed by incorporating EFRG score and various clinicopathological variables to estimate 1-, 3-, and 5-year overall survival (OS) probabilities. The “survivalROC” R package was used to generate time-dependent receiver operating characteristic (ROC) curves, and the area under the curve (AUC) was calculated to evaluate the predictive performance of the model at different time points. Calibration curves were generated using the “regplot” and “rms” R packages to assess the accuracy of the nomogram in predicting survival outcomes. Additionally, the “pec” R package was employed to plot concordance probability curves to evaluate the predictive precision of the EFRG score and clinical variables regarding patient survival.

### Tumor Mutation Burden Landscape and Drug Sensitivity Analysis

Drug sensitivity across subgroups was evaluated based on transcriptomic expression data using the “pRRophetic” R package, with IC50 values inferred from the Genomics of Drug Sensitivity in Cancer (GDSC) database. Somatic mutation data were obtained and preprocessed from the TCGA database using a Perl script. The mutation landscape was visualized using waterfall plots generated by the “maftools” R package to display the mutation frequency of key genes across different subgroups.

### Cell Culture

Human adult lightly pigmented epidermal melanocytes (HEMa-LP) and human malignant melanoma A375 cells were obtained from the Cell Bank of the Chinese Academy of Sciences (Shanghai, China). HEMa-LP cells were cultured in Medium 254 (Gibco, USA) supplemented with 1% Human Melanocyte Growth Supplement (HMGS, Gibco, USA). A375 cells were cultured in Dulbecco's Modified Eagle Medium (DMEM, high glucose; Gibco, USA) supplemented with 10% fetal bovine serum (FBS, Gibco, USA) and 1% penicillin-streptomycin (Gibco, USA). All cells were maintained in a humidified incubator at 37°C with 5% CO₂. Cells were passaged at a 1:3 ratio upon reaching 80-90% confluence, and the culture medium was replaced every 2-3 days.

### Western Blot Analysis

Western blotting was conducted to assess TTYH3 protein expression levels in HEMa-LP and A375 cells. Cells at logarithmic growth phase were harvested and lysed with RIPA buffer (Beyotime, China) supplemented with 1% protease inhibitor cocktail (Roche, Switzerland) on ice for 30 minutes. Lysates were centrifuged at 12,000 rpm for 15 minutes at 4 °C, and the supernatants were collected to determine protein concentrations using the BCA protein assay kit (Thermo Fisher Scientific, USA). Equal amounts of protein (30 μg) from each sample were separated via SDS-PAGE and transferred onto PVDF membranes (Millipore, USA). Membranes were blocked with 5% non-fat milk in TBST for 1 hour at room temperature and then incubated overnight at 4 °C with the primary antibody against TTYH3 (1:1000, Cat#24707-1-AP, Proteintech, USA). After washing, membranes were incubated with HRP-conjugated goat anti-rabbit secondary antibody (1:5000, Proteintech, USA) for 1 hour at room temperature. Protein bands were visualized using enhanced chemiluminescence (ECL) reagents (Thermo Fisher Scientific, USA) and imaged using the Bio-Rad imaging system. GAPDH (1:5000, Cat#ab181602, Abcam, UK) was used as an internal control. Band intensities were quantified using ImageJ software to compare the relative expression levels of TTYH3 between the two cell lines.

### TTYH3 Gene Knockdown

To investigate the functional role of TTYH3 in A375 melanoma cells, gene silencing was performed using small interfering RNA (siRNA) targeting TTYH3. Both TTYH3-specific siRNA and negative control siRNA (si-NC) were synthesized by GenePharma (Shanghai, China). Cells were seeded into 6-well plates and transfected using Lipofectamine™ 3000 (Invitrogen, USA) when cell density reached 50-60%, according to the manufacturer's protocol. A final concentration of 50 nM siRNA was used per well, and cells were incubated in antibiotic-free medium for 6 hours before replacing with complete medium containing 10% FBS. After 48 hours, cells were harvested for Western blot analysis to validate TTYH3 knockdown efficiency. All experiments were conducted in parallel with si-NC and si-TTYH3 groups and repeated at least three times to ensure reproducibility.

### CCK-8 Assay

Cell viability was assessed using the Cell Counting Kit-8 (CCK-8, Dojindo, Japan) following si-TTYH3 transfection in A375 cells for 48 hours. Transfected cells were seeded in 96-well plates in triplicate, and 10 μL of CCK-8 reagent was added to each well at 24, 48, 72, and 96 hours. Plates were incubated at 37 °C for 2 hours, and absorbance was measured at 450 nm using a microplate reader (BioTek, USA). Blank, si-NC, and si-TTYH3 groups were set up, and experiments were repeated three times. Differences in OD values across time points were analyzed to determine the impact of TTYH3 knockdown on cell proliferation.

### Colony Formation Assay

Following siRNA transfection, A375 cells were seeded into 6-well plates at a density of 500 cells/well and cultured for 10-14 days. When visible colonies formed, cells were washed with PBS, fixed in 4% paraformaldehyde for 15 minutes, and stained with 0.1% crystal violet for 30 minutes. Colonies were washed with water, air-dried, photographed, and counted using ImageJ software. Each group included three replicate wells, and the mean number of colonies was calculated.

### Transwell Migration

Cell migration ability following TTYH3 knockdown was assessed using Transwell chambers (8.0 μm pore size, Corning, USA). A total of 2×10⁴ transfected A375 cells in serum-free medium were added to the upper chamber, and the lower chamber was filled with medium containing 10% FBS as a chemoattractant. After 24 hours of incubation, non-migrated cells were removed, and the migrated cells were fixed with 4% paraformaldehyde for 15 minutes and stained with 0.1% crystal violet for 30 minutes. Five random fields per chamber were selected under a microscope for cell counting, and the average number of migrated cells was calculated.

### Statistical Analysis

All statistical analyses and visualizations were conducted using R software (version 4.4.1), Perl, and GraphPad Prism 9.0 (USA). Kaplan-Meier analysis and log-rank tests were performed to evaluate survival differences. Wilcoxon rank-sum test and Student's t-test were used for two-group comparisons, while ANOVA was used for multiple group comparisons. Multiple testing correction was applied as appropriate. All tests were two-sided, and *p*-values < 0.05 were considered statistically significant.

## Results

### Identification of Differentially Expressed Genes and Trait Related Gene Modules

**Figure 1:**
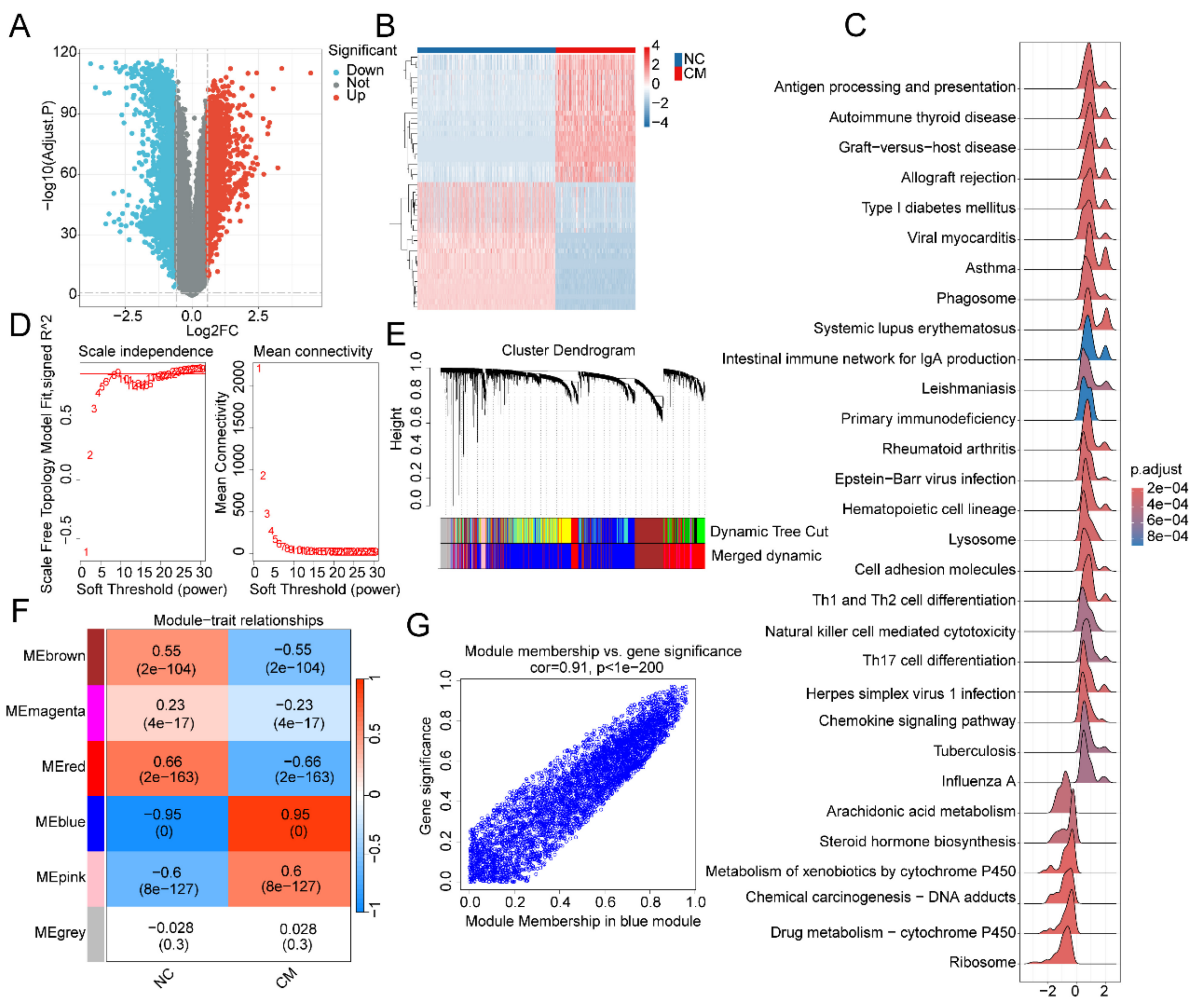
** Differential expression analysis and identification of trait-associated gene modules.** (A, B) Differential gene expression analysis between the NC and CM groups, with a threshold of |fold change| ≥ 1.5 and *p.*adjust < 0.05. Blue represents down-regulated genes, while red indicates up-regulated genes. (C) GSEA enrichment analysis. (D) Identification of the optimal soft threshold (β) and construction of the free network. (E) Integration of similar gene modules using dynamic tree cut. (F) Correlation analysis between clinical traits and gene modules. (G) Scatter plot analysis of the correlation between module membership and gene significance.

To ensure a clear analytical framework, our study was designed as a stepwise workflow integrating molecular characterization, subtype identification, prognostic modeling, and functional interpretation. A total of 813 NC samples and 447 CM samples were collected from the TCGA and Genotype-Tissue Expression project (GTEx) databases for subsequent analyses. Differential expression analysis was conducted using a threshold of |fold change| ≥ 1.5 and adjusted *p* < 0.05, resulting in the identification of DEGs between the NC and CM groups (Figure 1A, B). GSEA analysis indicated that immune-related signaling pathways such as primary immunodeficiency, cell adhesion molecules, Th1 and Th2 cell differentiation, natural killer cell-mediated cytotoxicity, and chemokine signaling pathway were significantly upregulated in the CM group. In contrast, pathways such as arachidonic acid metabolism, steroid hormone biosynthesis, and ribosome were significantly enriched in the NC group (Figure 1C). To identify gene modules associated with clinical traits, we constructed a WGCNA model using a soft threshold power of β = 8 (Figure 1D). A total of six distinct gene modules were identified through dynamic tree cut clustering (Figure 1E). Correlation analysis revealed significant associations between the six modules and clinical traits. Notably, the blue module was negatively correlated with the NC group (r = -0.95, *p* = 0) and positively correlated with the CM group (r = 0.95, *p* = 0), suggesting it is the module most strongly associated with clinical phenotypes (Figure 1F). Furthermore, a scatter plot demonstrated a strong correlation between gene significance and module membership within the blue module (Figure 1G).

### Identification of Differentially Expressed Efferocytosis-Related Gene Signatures and Mutational Landscape

**Figure 2:**
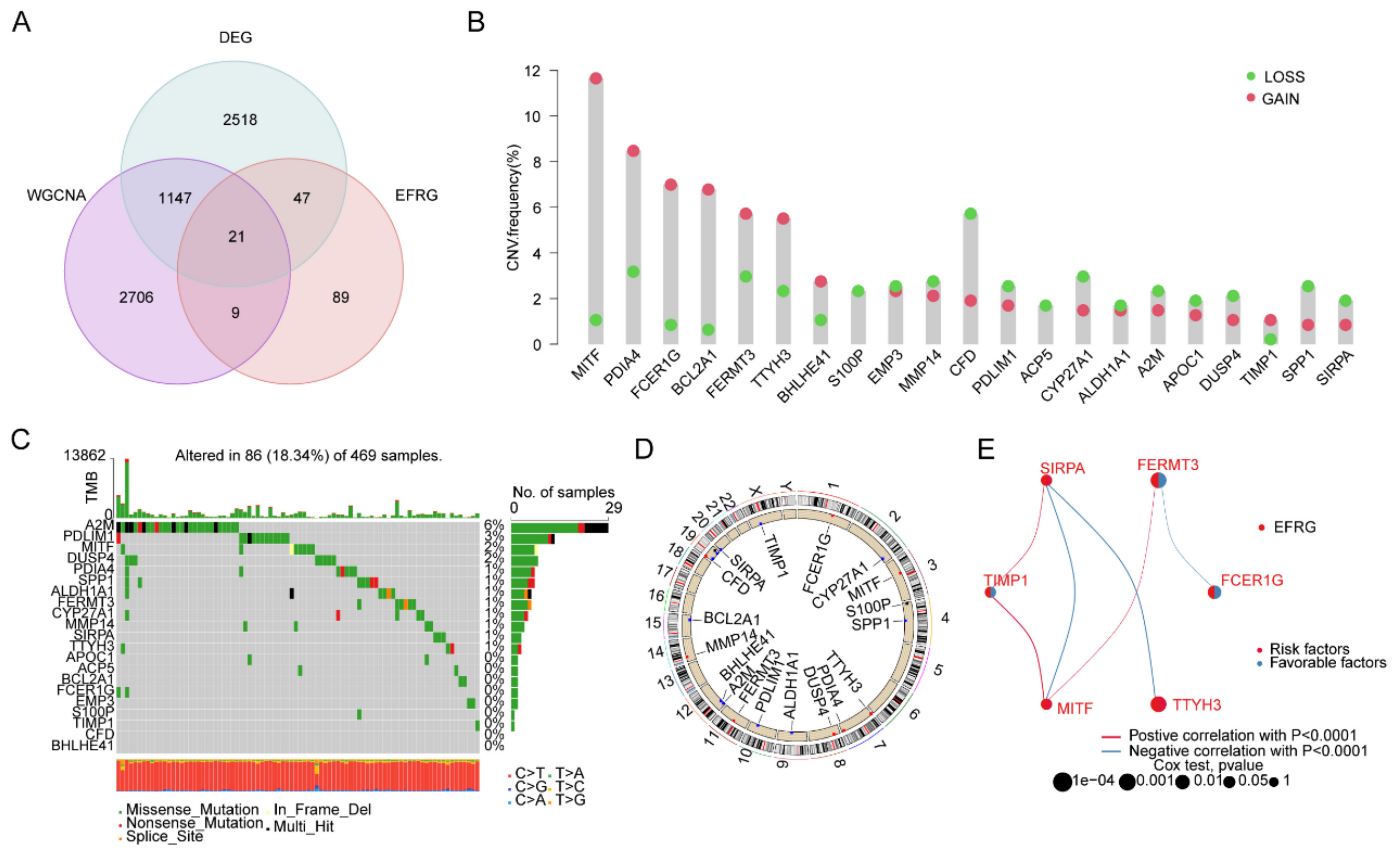
** Identification of DE-EFRG signature and mutation burden analysis.** (A) Identification of DE-EFRG signature associated with clinical traits using Venn diagram analysis. (B) CNV frequency analysis of the DE-EFRG signature. (C) Mutation burden landscape of DE-EFRG. (D) Co-localization analysis of the DE-EFRG signature on chromosomes. (E) Prognostic network analysis of the DE-EFRG signature.

By intersecting DEGs with predefined EFRGs and further filtering genes located in clinically significant WGCNA modules, followed by univariate Cox regression analysis (*p* < 0.05), we ultimately identified 21 prognostically relevant DE-EFRGs (Figure 2A). CNV analysis revealed that most DE-EFRG signatures exhibited copy number amplification in CM samples, including *MITF*, *PDIA4*, *FCER1G*, *BCL2A1*, *FERMT3*, and *TTYH3*, while *CFD* displayed notable CNV deletion (Figure 2B). The mutation landscape analysis showed that the overall mutation frequency of DE-EFRG signatures in CM samples was 18.34%, with A2M (6%), PDLIM1 (3%), MITF (2%), and DUSP4 (2%) exhibiting the highest mutation rates (Figure 2C). Chromosomal localization analysis further illustrated the co-localization of the 21 DE-EFRG signatures across different chromosomal regions (Figure 2D). Finally, an integrated gene interaction and prognostic network was constructed to assess the intergenic correlations and survival implications. The results revealed that *SIRPA*, *MITF*, and *TTYH3* were associated with poor prognosis in CM patients, whereas *TIMP1*, *FERMT3*, and *FCER1G* were correlated with favorable clinical outcomes (Figure 2E).

### Identification of EFRG Molecular Subtypes and Immune Microenvironment Landscape in CM

**Figure 3:**
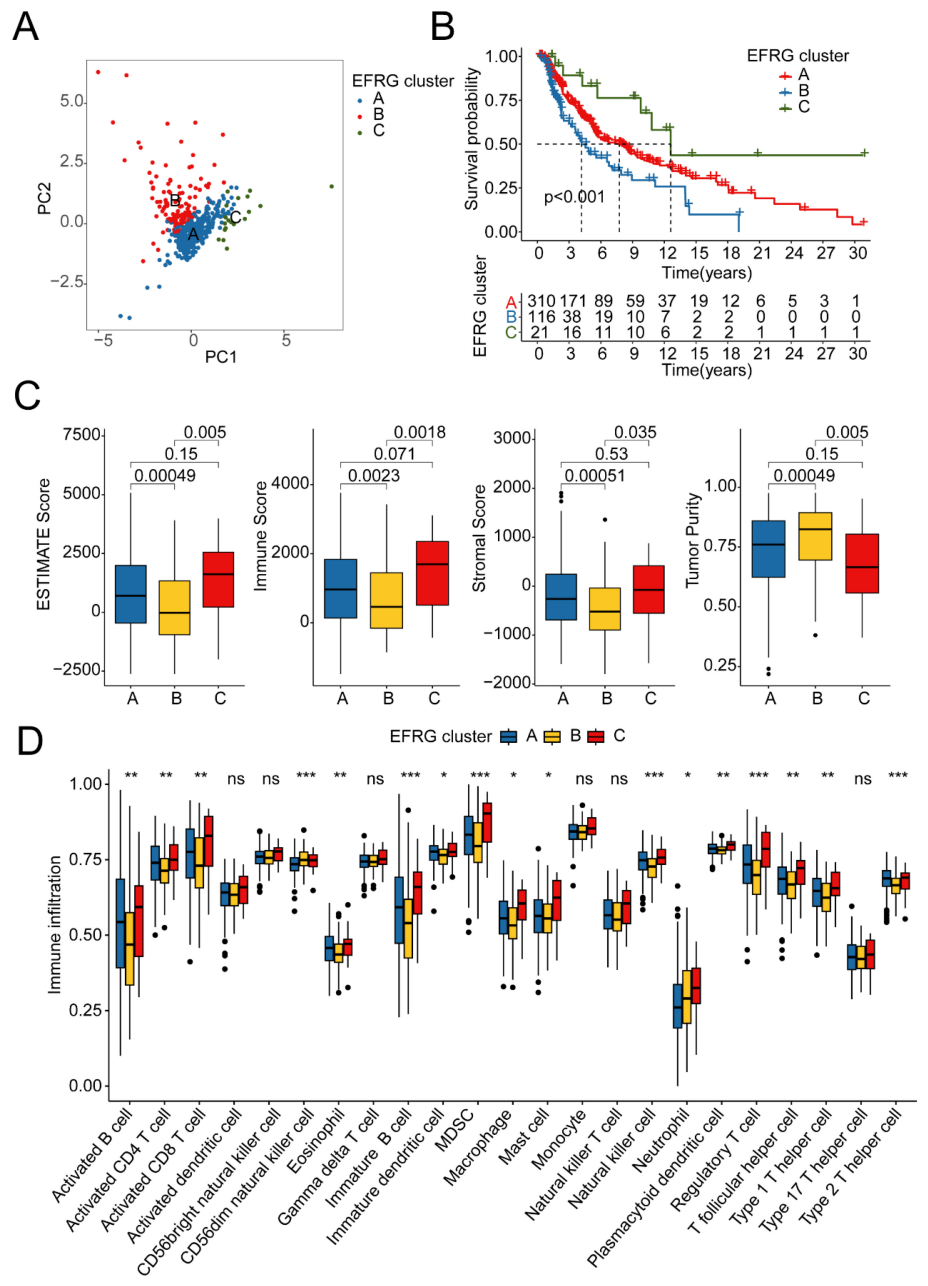
** Identification of EFRG molecular subtypes and immune microenvironment infiltration landscape.** (A) PCA plot showing the distribution characteristics of the three EFRG molecular subtypes. (B) Clinical prognostic survival analysis of EFRG molecular subgroups. (C) Immune infiltration status analysis based on the ESTIMATE algorithm. (D) ssGSEA analysis revealing the infiltration proportions of 23 immune cell types across EFRG molecular subgroups. Statistical significance: ns, not significant; *P < 0.05; **P < 0.01; ***P < 0.001.

We first identified DE-EFRGs between tumor and normal tissues to capture key molecular alterations associated with efferocytosis in CM. Given the potential heterogeneity of efferocytosis activity across tumors, we next performed consensus clustering based on DE-EFRGs to identify distinct molecular subtypes with differential efferocytosis patterns. According to the optimal parameters derived from the consensus clustering model, three distinct EFRG molecular subtypes were identified: subtype A (310 samples), subtype B (116 samples), and subtype C (21 samples). PCA demonstrated clear separation among the three EFRG subtypes, indicating distinct transcriptional patterns (Figure 3A). Survival analysis revealed that patients in EFRG subtype C exhibited significantly better overall survival outcomes compared to those in subtypes A and B, suggesting that subtype C is associated with favorable clinical prognosis (Figure 3B). ESTIMATE algorithm analysis showed that subtype B, which had the poorest prognosis, was characterized by significantly lower ESTIMATE score, immune score, and stromal score, along with markedly higher tumor purity, implying an immunosuppressive TME (Figure 3C). ssGSEA indicated that the infiltration levels of immune cells such as activated B cells, activated CD4^+^ T cells, immature B cells, immature dendritic cells, MDSC, and activated CD8^+^ T cells were significantly decreased in subtype B. In contrast, infiltration of CD56 dim natural killer cells was notably increased (Figure 3D).

### Construction of the EFRG Scoring System Using Machine Learning and Prognostic Independence Assessment

**Figure 4:**
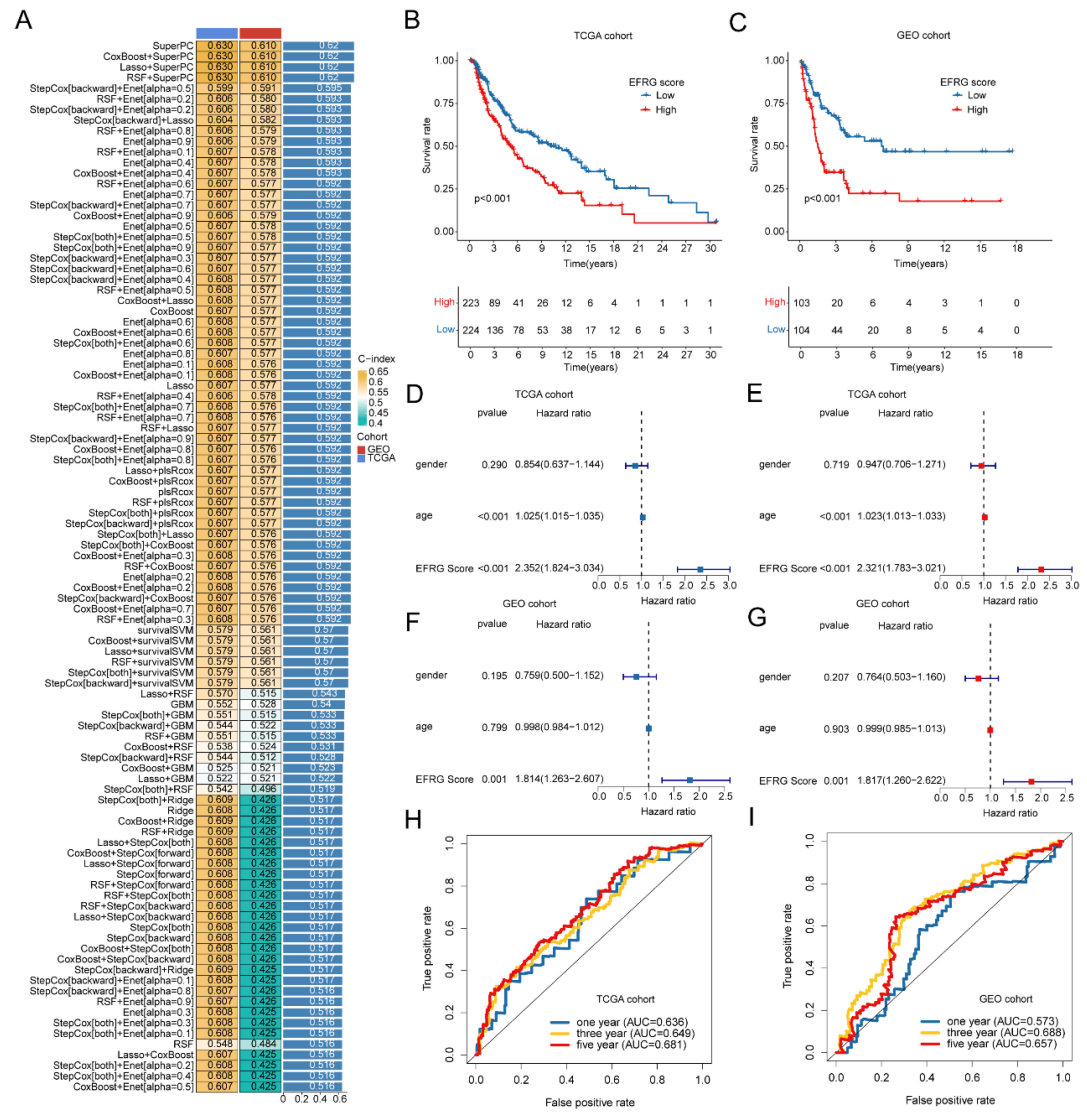
** Construction of the EFRG scoring system based on machine learning algorithms and evaluation of independent prognostic value.** (A) Consistency index calculation of 101 algorithm combinations from 10 machine learning methods in the TCGA and GEO cohorts. (B, C) Clinical prognostic survival analysis in the TCGA and GEO cohorts. (D-G) Univariate and multivariate Cox analysis based on clinical pathological variables and EFRG score index in the TCGA and GEO cohorts. (H, I) Time-dependent ROC curve analysis for 1-year, 3-year, and 5-year survival predictions.

In this study, molecular subtypes and the EFRG score were derived through sequential but conceptually distinct analyses. Specifically, consensus clustering based on DE-EFRGs was first performed to capture population-level heterogeneity in efferocytosis patterns. Subsequently, an individual-level prognostic scoring system was constructed using machine learning algorithms based on the same pool of DE-EFRGs. Thus, molecular subtypes reflect global transcriptional patterns, whereas the EFRG score provides a quantitative measure of individual patient risk. To evaluate the clinical relevance of the identified efferocytosis-based subtypes, we performed survival analysis to determine whether these molecular patterns were associated with patient prognosis. Utilizing both the TCGA training cohort and the GSE65904 validation cohort, we assessed 101 combinations of 10 machine learning algorithms under a LOOCV framework. Ultimately, four optimal algorithm combinations were identified: SuperPC, CoxBoost+SuperPC, Lasso+SuperPC, and RSF+SuperPC (Figure 4A). A multivariate Cox regression model was constructed to calculate the EFRG risk score and evaluate its prognostic significance. Kaplan-Meier survival analysis demonstrated that patients in the low EFRG score subgroup had significantly improved survival outcomes in both the TCGA training set and GSE65904 validation set, compared to those in the high subgroup (Figure 4B, C).

Moreover, univariate and multivariate Cox regression analyses confirmed that the EFRG score was significantly associated with poor prognosis in both cohorts (HR > 1, *p* < 0.05), suggesting that the EFRG score serves as an independent prognostic indicator for CM (Figure 4D-G). Time-dependent ROC curves further validated the model's predictive performance, with AUC of 0.636, 0.649, and 0.681 for 1-, 3-, and 5-year survival in the TCGA cohort, and 0.573, 0.688, and 0.657, respectively, in the GEO cohort (Figure 4H, I). Collectively, these results indicate that the EFRG-based prognostic model enables accurate risk stratification of CM patients and can robustly predict clinical outcomes as an independent prognostic biomarker.

### Nomogram Construction Based on Clinicopathological Features and EFRG Score

**Figure 5:**
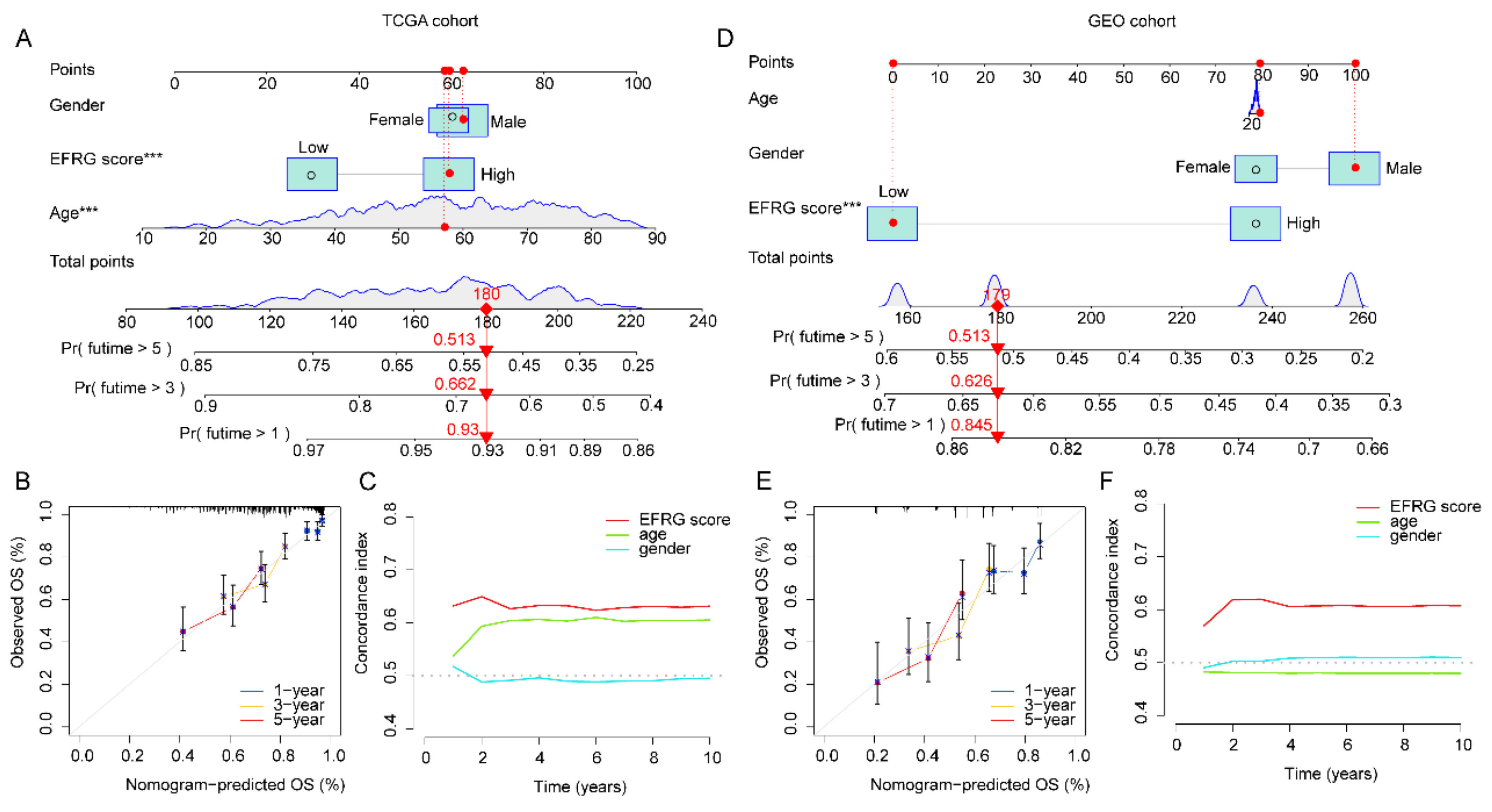
** Construction of the nomogram model.** (A) Construction of the nomogram model based on clinical pathological variables and EFRG score index in the TCGA cohort. (B) Calibration curve analysis of the nomogram model in the TCGA cohort. (C) C-index curve analysis in the TCGA cohort. (D) Construction of the nomogram model based on clinical pathological variables and EFRG score index in the GEO cohort. (E) Calibration curve analysis of the nomogram model in the GEO cohort. (F) C-index curve analysis in the GEO cohort.

Based on clinicopathological variables and the EFRG scoring system, we constructed a nomogram model to predict 1-, 3-, and 5-year overall survival probabilities in CM patients within the TCGA and GEO cohorts (Figure 5A, D). Calibration plots demonstrated strong concordance between the predicted and actual survival probabilities at 1, 3, and 5 years in both independent cohorts, indicating good predictive accuracy of the nomogram (Figure 5B, E). Furthermore, the C-index analysis revealed that the EFRG score exhibited superior prognostic performance compared to traditional clinicopathological variables, highlighting its potential clinical utility in CM risk prediction (Figure 5C, F).

### Immune Infiltration Landscape in EFRG-Based Risk Subgroups

**Figure 6:**
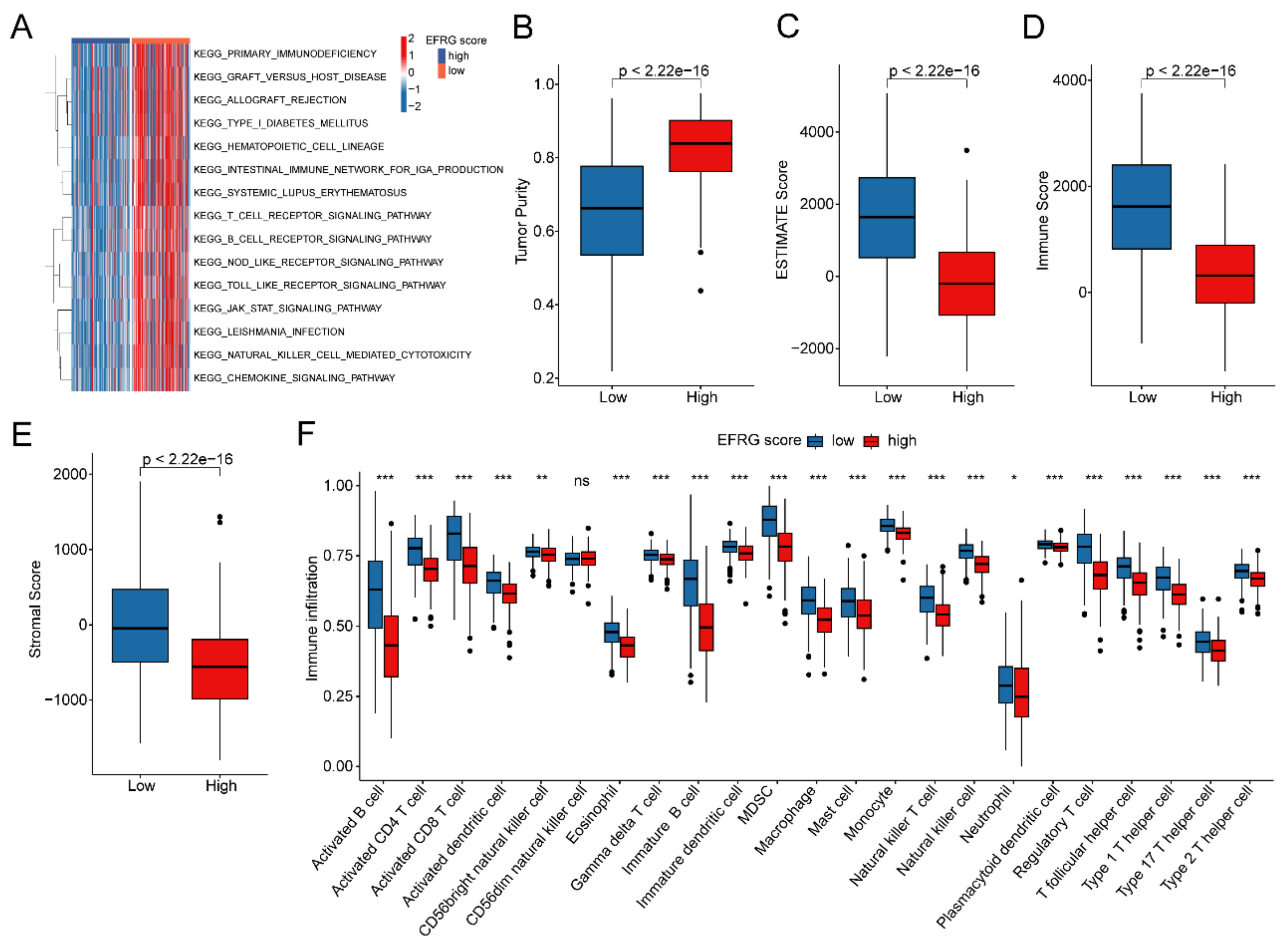
** Immune microenvironment infiltration landscape analysis of EFRG score subgroups.** (A) Differential analysis of KEGG signaling pathways across EFRG score subgroups. (D-E) Immune infiltration status analysis based on the ESTIMATE algorithm. (F) ssGSEA analysis revealing the infiltration proportions of 23 immune cell types across EFRG score subgroups. Statistical significance: ns, not significant; *P < 0.05; **P < 0.01; ***P < 0.001.

To comprehensively characterize the tumor immune microenvironment, multiple complementary algorithms were applied, which provide complementary insights into immune composition, functional activity, and microenvironmental context. To further investigate the biological differences between EFRG-based risk subgroups, we performed GSVA to explore differential regulation of KEGG signaling pathways. The results indicated significant upregulation of several immune-related pathways in the low EFRG score group, including the T cell receptor signaling pathway, B cell receptor signaling pathway, NOD-like receptor signaling pathway, and natural killer cell-mediated cytotoxicity (Figure 6A). ESTIMATE analysis showed that the low-risk subgroup exhibited significantly higher immune scores, stromal scores, and ESTIMATE scores, alongside lower tumor purity, suggesting an immune-enriched microenvironment in these samples (Figure 6B-E). Consistent with this, ssGSEA analysis revealed significantly elevated infiltration levels of various immune cell types in the low EFRG score group, including activated B cells, activated CD4^+^ T cells, activated CD8^+^ T cells, and activated dendritic cells, indicating a robust immune-activated phenotype (Figure 6F).

### Immunotherapy Response and Drug Sensitivity Analysis

**Figure 7:**
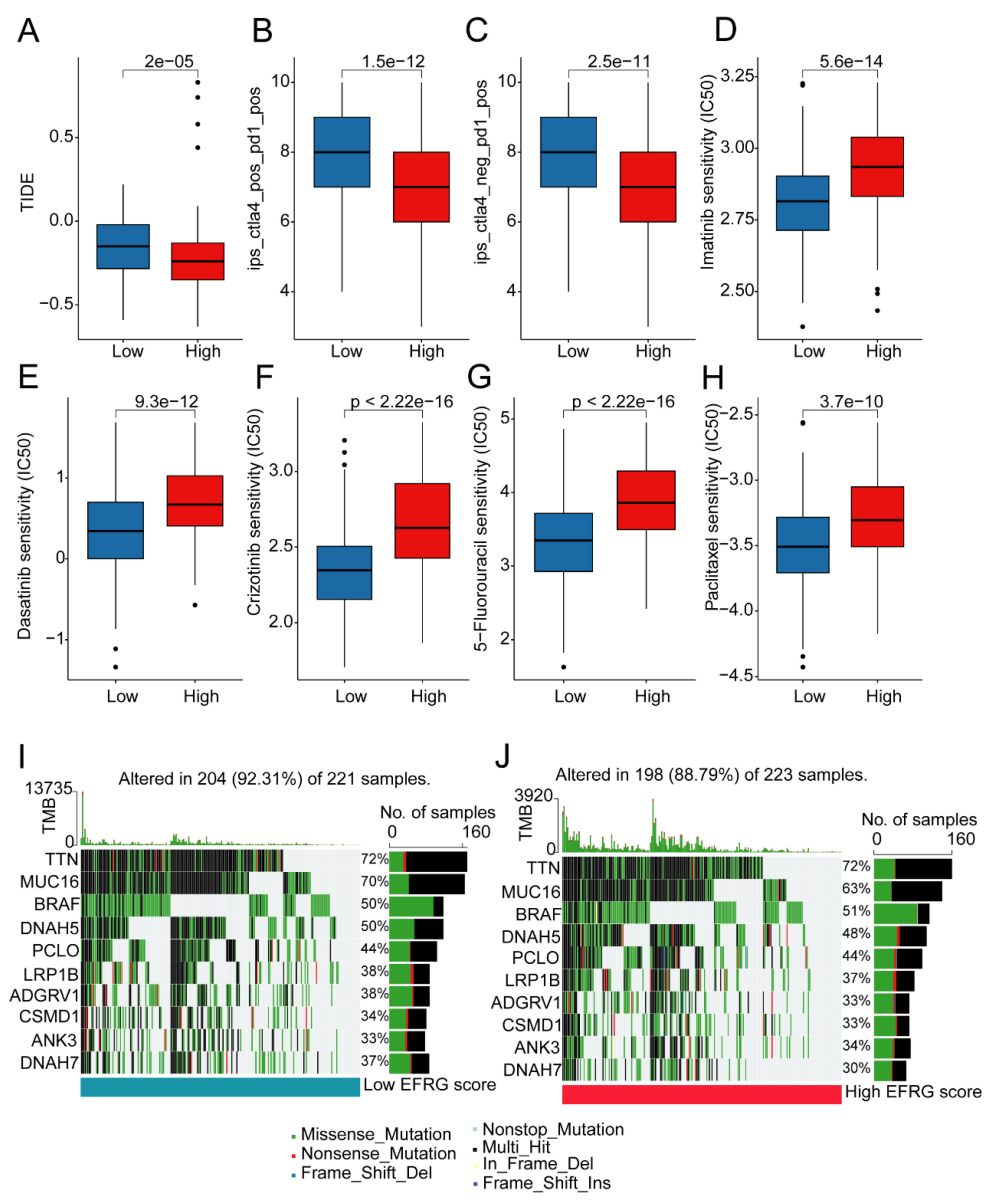
** Prediction of immune therapy response and drug sensitivity analysis.** (A) TIDE score analysis. (B, C) IPS score analysis across EFRG score subgroups. (E-H) Prediction of drug sensitivity with IC50 values. (I, J) Mutation burden landscape across EFRG score subgroups.

Subsequently, we further evaluated the potential response to immunotherapy across different EFRG score subgroups. The TIDE score results indicated a significant increase in the TIDE score in the low EFRG score subgroup, suggesting a higher likelihood of immune escape during immunotherapy in this subgroup (Figure 7A). IPS analysis revealed that the low EFRG score subgroup benefited more from clinical treatment when treated with CTLA-4 and PD1 inhibitors (Figures 7B, C). Additionally, drug sensitivity analysis showed significantly lower IC50 values for 5-Fluorouracil, Imatinib, Dasatinib, Crizotinib, and Paclitaxel in the low EFRG score subgroup, suggesting potentially better therapeutic responses to these drugs (Figures 7D-H). The mutation burden analysis revealed a higher somatic mutation rate in the low EFRG score subgroup compared to the high EFRG score subgroup, with MUC16 at 70% and DNAH7 at 37%, among others (Figures 7I, J).

### Knockdown of TTYH3 Expression Significantly Inhibits CM Proliferation and Migration

**Figure 8:**
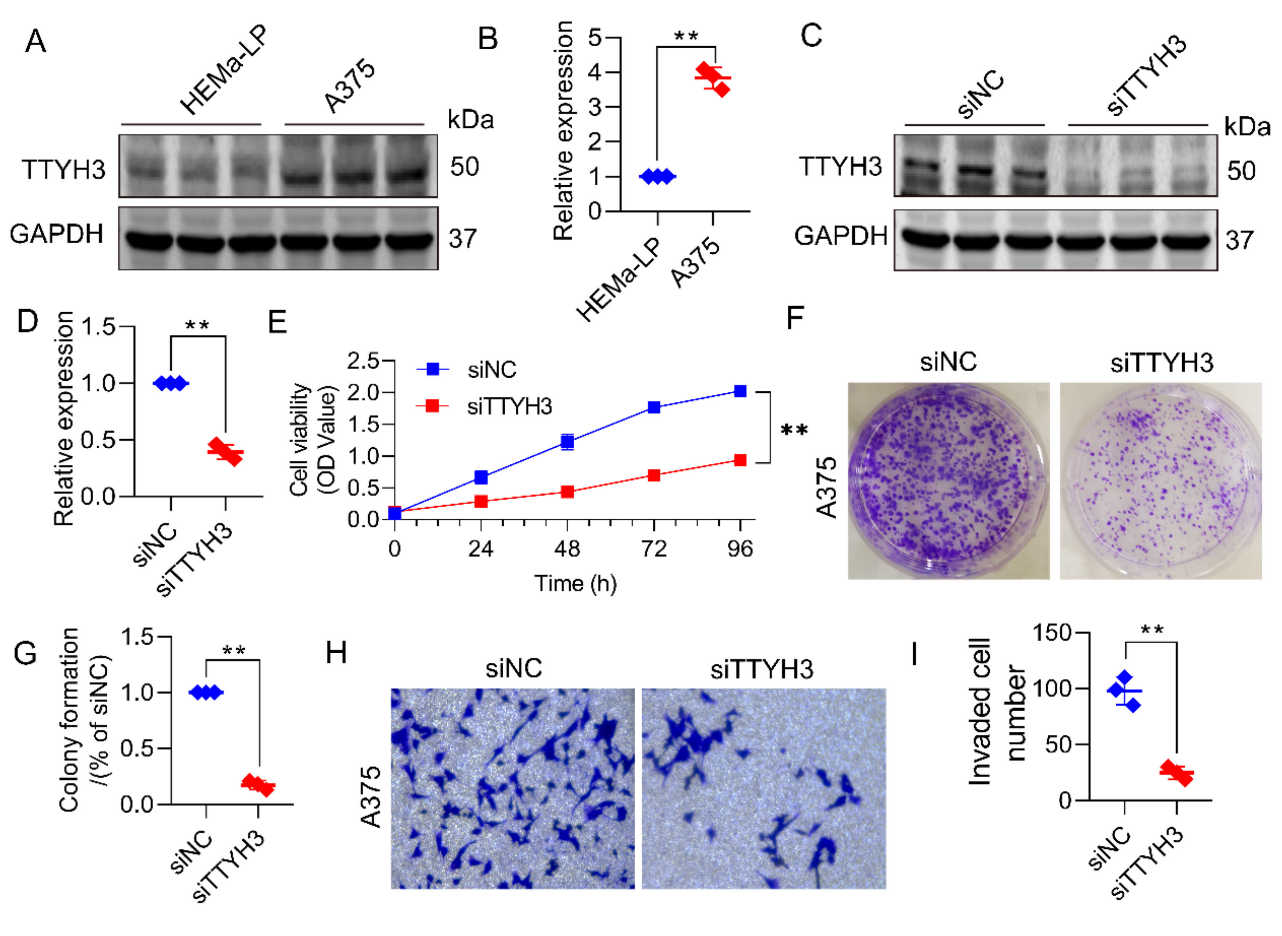
** Knockdown of TTYH3 expression significantly inhibits CM cell proliferation and migration.** (A) Western blot analysis of TTYH3 expression in HEMa-LP and A375 cell lines (n=3). (B) Quantitative analysis of TTYH3 protein expression (n=3). (C, D) Validation of siTTYH3 knockdown efficiency by Western blot analysis (n=3). (E) CCK8 assay revealing the cell viability after siTTYH3 interference (n=3). (F-I) Colony formation assay and Transwell migration analysis (n=3). Statistical significance: *p<0.05; **p<0.01; ***p<0.001.

In the construction of the EFRG scoring system, we found that *TTYH3* had the highest risk coefficient, suggesting that it might be a key target associated with CM prognosis. Western blot analysis revealed a significant increase in TTYH3 protein expression in the A375 cell line, compared to the control HEMa-LP cell line (Figures 8A, B, *p* <0.05). To further investigate the potential role of TTYH3 in CM development, we used siRNA interference technology to construct the siTTYH3 model in the A375 cell line. The interference efficiency results indicated a significant reduction in TTYH3 protein expression in the siTTYH3 group compared to the siNC group (Figures 8C, D). CCK8 assay results demonstrated that silencing TTYH3 significantly inhibited cell viability in the A375 cell line (Figure 8E). Moreover, colony formation and invasion assays showed that TTYH3 knockdown significantly inhibited A375 cell proliferation and migration (Figures 8F-I). Based on these findings, we conclude that inhibiting TTYH3 expression significantly suppresses CM proliferation and migration, further clarifying its role in CM development.

## Discussion

The role of efferocytosis in cancer remains incompletely understood. Depending on tumor type and stage, efferocytosis has been reported to exhibit both anti-tumor and pro-tumor effects 26. Our findings highlight the prognostic value and biological significance of the efferocytosis process in CM. Preliminary evidence has already suggested a role for efferocytosis in melanoma. Receptor tyrosine kinases, which play critical roles in efferocytosis, are aberrantly expressed in melanoma 24. In murine melanoma models, a neutrophils secreted efferocytosis-related protein, annexin A1, has been shown to promote melanoma metastasis 27. Moreover, depletion of mesenchymal stromal cells (MSCs) in mice can enhance macrophage-mediated efferocytosis, inducing pathological angiogenesis and immune suppression, which suggests that inhibition of efferocytosis may serve as a promising strategy to restore anti-tumor immunity 28. However, due to the dual nature of efferocytosis, some studies have also shown that an efferocytosis facilitating chemokine, CX3CL1, is associated with anti-tumor immune responses in melanoma 29. Nonetheless, current research remains largely limited to murine models. Although our study identifies potential efferocytosis-related targets in melanoma patients, the precise effects and mechanisms of efferocytosis in human melanoma warrant further investigation due to the complex and context-dependent nature of this process.

To further interpret these findings mechanistically, it is important to interpret our results within a mechanistic framework linking efferocytosis, immune regulation, and therapeutic response in melanoma. Efferocytosis represents a critical immunological checkpoint in the tumor microenvironment, as it governs the clearance of apoptotic tumor cells and shapes downstream immune responses 30. In melanoma, a tumor characterized by high immunogenicity and substantial cell turnover, increased efferocytosis activity may lead to continuous removal of dying tumor cells in an immunologically silent manner, thereby limiting the release of DAMPs and reducing effective antigen presentation 31. This process may promote immune tolerance by driving macrophage polarization toward an M2-like phenotype and enhancing the production of anti-inflammatory cytokines such as IL-10 and TGF-β 32. Consequently, tumors with elevated efferocytosis-related signatures may exhibit an immunosuppressive microenvironment despite the presence of immune cells. Conversely, reduced or dysregulated efferocytosis may allow accumulation of apoptotic debris, triggering pro-inflammatory signaling and enhancing anti-tumor immunity 8. This bidirectional role provides a plausible explanation for the distinct immune infiltration patterns and survival outcomes observed between different EFRG-based subgroups in our study.

Our results demonstrate that patients with lower levels of immune infiltration exhibit poorer prognoses. Interestingly, in addition to key anti-tumor immune components including CD4^+^ and CD8^+^ T cells, myeloid-derived suppressor cells (MDSCs) and regulatory T cells (Tregs) were found to be less abundant in CM patients with worse outcomes. It is generally believed that the progression and decreased overall survival of melanoma are associated with increased quantity and functionality of Tregs and MDSCs 33, 34. This apparent contradiction warrants careful consideration. First, potential inaccuracies inherent to computational immune infiltration estimation methods cannot be excluded. Second, it is possible that high-risk patients exhibit an immune-deficient or advanced immune-escape phenotype, characterized by reduced chemokine production, vascular abnormalities, or antigen loss, leading to globally diminished immunogenicity and a broad decline in diverse immune cell populations, including immunosuppressive subsets 35. Third, the composition of MDSCs may differ across risk groups: polymorphonuclear MDSCs (PMN-MDSCs), which are less immunosuppressive than monocytic MDSCs (M-MDSCs), could dominate the TME in high-risk patients, thereby reducing the overall immunosuppressive burden as measured by bulk signatures 36, 37. Additionally, Tregs infiltration level has been associated with sensitivity to immunotherapy, which may also explain the reduced Treg infiltration difference by risk stratification 38-40. From the perspective of efferocytosis, we hypothesize that impaired efferocytosis in high-risk tumors leads to accumulation of secondary necrotic debris, which triggers atypical pro-inflammatory signals that paradoxically fail to recruit or sustain typical immunosuppressive cells while still fostering an immunosuppressive metabolic milieu. Conversely, efficient efferocytosis in low-risk tumors promotes apoptotic cell clearance without releasing DAMPs, thereby maintaining an organized immune architecture that supports effector T cell infiltration and balanced regulatory components. This interpretation aligns with the established role of efferocytosis in preventing necrosis-associated inflammation and preserving immune homeostasis 7, 9. Future studies employing single-cell and spatial transcriptomics are needed to dissect the functional states of immune cells in EFRG-defined subgroups and to validate these hypotheses.

Our *in vitro* experiments confirmed the oncogenic role of TTYH3 in melanoma cells, promoting cell proliferation, invasion, and colony formation. TTYH3 is the third member of the Tweety homolog (TTYH) family in mammals and encodes a gated chloride channel involved in various cellular processes 41, 42. Upregulation of TTYH3 has been observed in multiple cancer types, suggesting a potential role in tumorigenesis 43, 44. Several mechanisms have been proposed for TTYH3-mediated tumor progression. TTYH3 has been shown to activate the Wnt/β-catenin or FGFR1/MEK/ERK signaling pathways to promote tumor development 45, 46. It can also facilitate tumor malignancy by modulating tumor-associated macrophage polarization 47. Additionally, TTYH3 has been implicated in promoting epithelial-mesenchymal transition (EMT), metastasis, and angiogenesis in colorectal cancer via regulation of histone deacetylase 7 44. While our current results did not clarify the exact pathways through which TTYH3 exerts its effects in melanoma cells, these previously reported mechanisms suggest valuable directions for future mechanistic investigations 48-50. Given that TTYH3 is a chloride channel implicated in phagocyte function and cell volume regulation 41, 42, its contribution to melanoma progression may also involve modulation of efferocytosis efficiency within the TME. Although direct evidence is lacking, chloride flux is known to regulate phagosome maturation and apoptotic cell engulfment 51, raising the possibility that TTYH3 overexpression in tumor cells or tumor-associated macrophages could influence efferocytosis dynamics, thereby shaping immune suppression and tumor growth. Future studies should explore whether TTYH3-mediated chloride signaling intersects with efferocytosis related pathways in CM.

In recent years, ICIs has made significant advances in the treatment of CM and has become the first-line therapy for advanced melanoma 1, 4. However, the overall response rate remains limited, with only approximately 30% - 50% of patients achieving durable responses, and there is considerable interindividual variability 52. Moreover, immune-related adverse events (irAEs) can affect multiple organ systems, posing serious complications and increasing treatment risks and complexity 53. Therefore, the lack of effective biomarkers to predict immunotherapy response represents a major challenge in clinical practice, highlighting the urgent need for refined risk stratification systems 54, 55. Emerging evidence suggests that integrating patients' genomic features, immune microenvironment status, and multi-omics analysis can help identify subpopulations that are more likely to benefit from immunotherapy and have favorable prognoses 56, 57. Our results show an apparent discrepancy between two immunotherapy predictors in the EFRG-based risk model. Low-risk patients had higher IPS but also higher TIDE scores 58, 59. This contradiction can be reconciled by noting that IPS reflects baseline effector immune cell abundance, whereas TIDE integrates T cell dysfunction and exclusion 59. In our low-risk, immune-enriched tumors, high TIDE is likely driven by the dysfunction component rather than exclusion, as supported by elevated CD8⁺ T cell infiltration. Chronic antigen stimulation can induce reversible T cell exhaustion, which increases TIDE scores yet maintains sensitivity to PD-1/CTLA-4 blockade. Thus, the low-risk subgroup exhibits an “inflamed but functionally reversible” phenotype optimally poised for ICI benefit. From an efferocytosis perspective, efficient apoptotic cell clearance reduces chronic inflammation and prevents irreversible exhaustion 7, while allowing transient dysfunction captured by TIDE. These insights refine how EFRG-defined risk groups might guide personalized immunotherapy. Importantly, this apparent discrepancy between IPS and TIDE should be interpreted with caution. IPS primarily reflects the baseline abundance of effector immune cells, whereas TIDE integrates mechanisms of T cell dysfunction and exclusion. Therefore, the observed pattern in the low-risk subgroup may suggest a complex immune context characterized by both immune activation and potential dysfunction. One possible explanation is that increased TIDE scores in this group are driven by T cell dysfunction rather than exclusion, which may still be reversible under immune checkpoint blockade. Moreover, different immunotherapy prediction algorithms are based on distinct biological assumptions and training datasets, which may contribute to inconsistent results across methods. These findings are based on computational predictions and should be further validated in prospective clinical cohorts.

In conclusion, based on multi-omics data, this study systematically analyzed the role of EFRGs in CM using various bioinformatic approaches, constructed a prognostic model, and elucidated their significance in molecular mechanisms, immune microenvironment regulation, and survival prediction. These findings provide theoretical support and potential targets for personalized melanoma therapy. Nevertheless, several limitations should be acknowledged. First, the primary data were derived from public databases, which may introduce sample selection bias. Second, although multiple algorithms were applied for cross-validation to enhance the robustness of our results, independent cohorts and clinical samples are still lacking for validation. Moreover, our study did not deeply explore the molecular mechanisms through which EFRGs influence CM progression, necessitating further* in vivo* and *in vitro* functional studies. Future research should incorporate real-world datasets and experimental validation to improve the reliability and translational value of these findings.

## Supplementary Material

Supplementary table.

## Data Availability

The datasets used and analyzed during the current study are available from the corresponding author on reasonable request.
