# Supplementary material for: Comprehensive Characterization and Prognostic Modeling of Efferocytosis-Related Genes in Cutaneous Melanoma
Source: J Cancer. 2026 Jul 29;17(8):1419–34. doi: 10.7150/jca.130446 (PMC13455216; doi:10.7150/jca.130446)
Supplement: Supplementary file 1 [file jcav17p1419s1.pdf]

SupplementaryTable1. The list of efferocytosis-related genes.
S100P
TFF3
TESC
SAA1
SPP1
S100A9
IGFBP2
FOSB
MARCO
ALDH1A1
MMP9
LYZ
PLAU
CCL18
DUSP4
MRC1
BASP1
OLR1
HIST1H1C
VSIG4
VCAN
C1QB
CFD
APOC1
MCEMP1
C1QC
CAV1
CD52
C1QA
ITGB8
CYBB
FCGR3A
COLEC12
PER1
KLF4
CD163
IL6
RETN
CYP27A1
MNDA
MSR1
A2M
RBP4
ASPH
ODC1
CCL2
PLBD1
TYROBP
EMB
RGS16
MMP14
PPARG
OSCAR
BCL2A1
FABP5

SIGLEC1
SPI1
AQP9
HCK
ALOX5
PTAFR
SLCO2B1
MS4A7
FABP4
LY86
BHLHE41
FCER1G
MS4A4A
FPR1
SOCS3
TUBB6
CSTA
NCF2
RGCC
KCTD12
PAPSS2
BATF
HCST
CD37
LST1
THBD
FBP1
HBEGF
EMP1
CD83
C5AR1
FERMT3
SERPING1
FBXO32
SDS
ACP5
LAPTM5
IER5L
LAIR1
CD93
PCOLCE2
PILRA
SGK1
FGR
LIPA
VIM
PTGER4
IL1B
JUNB
FCN1
GLIPR2
EMP3
ITGAX
SLC15A3
C1orf162
SLC7A7

LBH
SAMHD1
CD36
SLC11A1
TLR4
SIRPA
TIMP1
DAB2
FAM49A
PMP22
TTYH3
CAT
ABCG1
THEMIS2
SLA
MMP19
STX11
ENG
PIK3IP1
ARRDC4
JUND
ACE
PECAM1
CCRL2
TNS3
PDIA4
EPB41L3
SH3BP5
CXCL16
ARHGAP18
EMILIN2
TTC39C
MOB3B
GPR65
IL3RA
SMAD7
QKI
C1orf56
RCAN1
PDLIM1
DOCK4
PDIA6
EREG
MATK
CD300E
MITF
OTUD1
GLDN
PNPLA6
CD68
LILRB3
SLC16A6
MCOLN1
SLC31A2
FRMD4A
IL1A
